# A case of metachronous liver metastasis in small intestinal gastrointestinal stromal tumor: real-world integration of targeted therapy and hepatectomy 14 years after initial resection

**DOI:** 10.1093/gastro/goaf059

**Published:** 2025-06-30

**Authors:** Youwen Fan, Zheng Yang, Kai Liu, Gang Deng, Di Tang

**Affiliations:** Department of General Surgery, The Seventh Affiliated Hospital, Sun Yat-sen University, Shenzhen, Guangdong, P. R. China; Department of Pathology, The Seventh Affiliated Hospital, Sun Yat-sen University, Shenzhen, Guangdong, P. R. China; Department of General Surgery, The Seventh Affiliated Hospital, Sun Yat-sen University, Shenzhen, Guangdong, P. R. China; Department of General Surgery, The Seventh Affiliated Hospital, Sun Yat-sen University, Shenzhen, Guangdong, P. R. China; Department of General Surgery, The Seventh Affiliated Hospital, Sun Yat-sen University, Shenzhen, Guangdong, P. R. China; Guangdong Provincial Key Laboratory of Digestive Cancer Research, The Seventh Affiliated Hospital of Sun Yat-sen University, Shenzhen, Guangdong, P. R. China

## Introduction

Intestinal gastrointestinal stromal tumor (GIST) is the most common mesenchymal tumor in the gastrointestinal tract, with an estimated incidence of 7–15 per million population [1]. Among resected GIST patients, approximately 40% will experience recurrence or metastasis, with the liver being the most common site. Small intestinal GIST has a higher propensity for liver metastasis. Liver metastasis typically occurs within 16–39 months [2], though it can take as long as 30 years [3]. In real-world settings, financial constraints may limit access to mutational testing, complicating the use of targeted therapies. Here, we report a case of small intestinal GIST with metachronous liver metastasis that was effectively managed through sequential tyrosine kinase inhibitor (TKI) therapy (imatinib followed by sunitinib) combined with elective hepatectomy and has achieved long-term survival of 14 years after initial GIST resection. While this empirical, response-guided treatment strategy—necessitated by financial constraints—offers a pragmatic approach in real-world settings, it carries the inherent risk of ineffective targeted therapy.

## Case report

**Figure 1.**
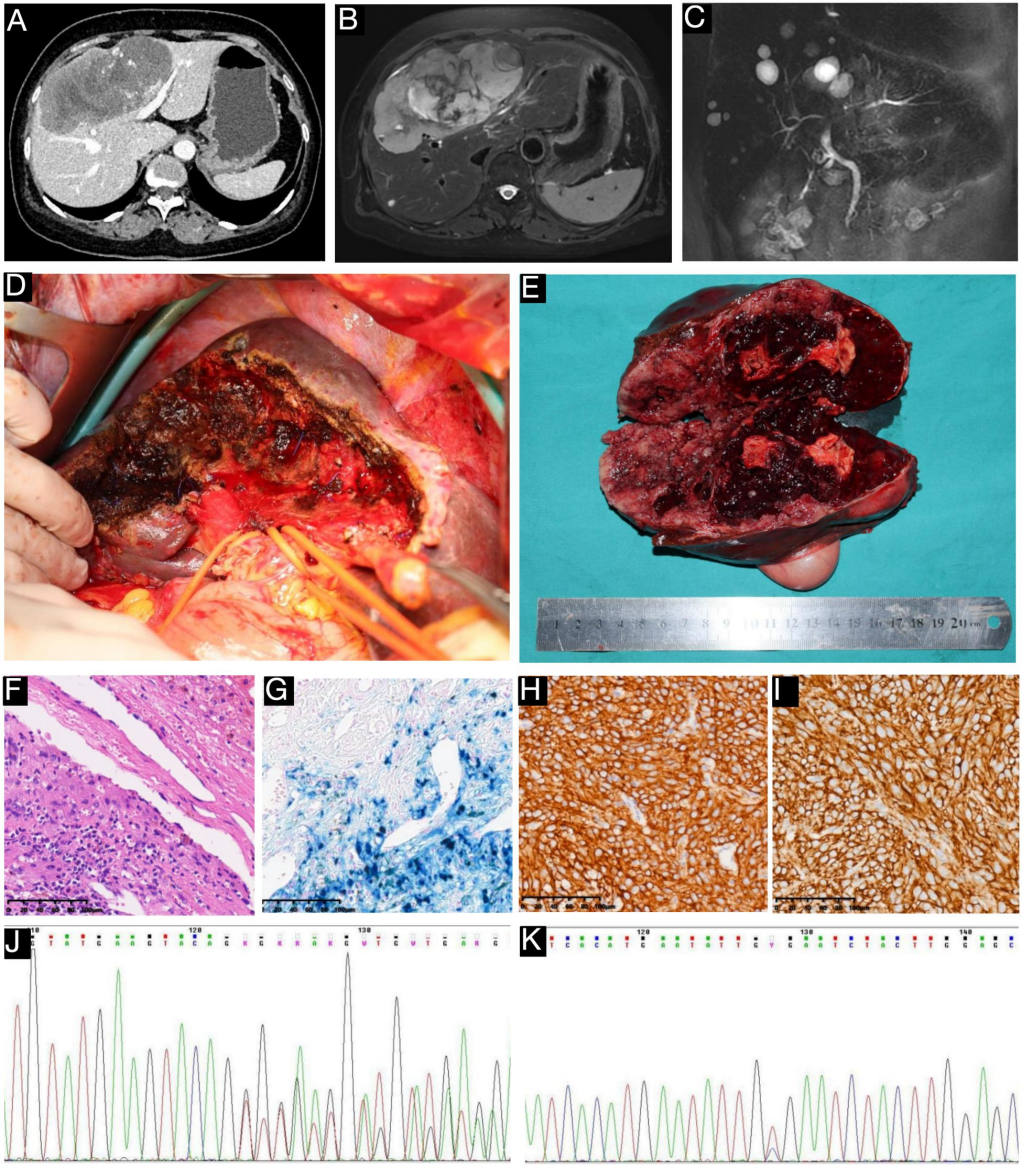
Preoperative imaging, surgical treatment, tumor pathology and mutational analysis. (A) A preoperative computed tomography scan shows a large mass in liver segments S4, S5, and S8. (B) A preoperative magnetic resonance imaging demonstrates a large mass abutting the porta hepatis. (C) Preoperative magnetic resonance cholangiopancreatography reveals hilar compression and narrowing of the left and right bile ducts. (D) An intraoperative image shows the residual liver after resection. (E) The gross tumor specimen is presented post-resection. (F–I) Histopathological characteristics of the tumor: (F): hematoxylin and eosin staining, ×20. (G) Prussian blue staining positive, ×20. (H) CD117-positive immunostaining, ×20. (I) DOG1-positive immunostaining, ×20. (J and K) Sanger sequencing results for mutational analysis: (J) C-KIT exon 11 (p.W557_K558del) mutation. (K) C-KIT exon 13 (p.V654A) mutation.

A 59-year-old female underwent radical resection of a small intestinal GIST 14 years ago (in 2011) at a local hospital. Postoperative pathology revealed a high-risk GIST, 7 cm in diameter, with a mitotic count of 6 per 50 high-power fields. No postoperative adjuvant imatinib therapy was administered as its use in the adjuvant setting was not yet widely adopted in 2011. Eleven years ago (in 2014), a 9-cm solitary liver metastasis was detected, and imatinib 600 mg/day was prescribed at that hospital, likely due to the large size of the metastasis, achieving a partial response with the lesion shrinking to 4 cm in diameter. Approximately 3.5 years ago (in September 2021), the patient presented to our hospital with an enlarging liver mass. On admission, computed tomography and magnetic resonance imaging revealed a 14-cm solitary tumor in Couinaud segments 4, 5, and 8, abutting the porta hepatis (Figure 1A–C). Following a multidisciplinary team discussion, limited disease progression during the imatinib response period was confirmed, and an open central hepatectomy was performed (in October 2021) to resect the malignancy (Figure 1D). The resected mass exhibited extensive hemorrhage, cystic degeneration, and necrosis (Figure 1E). Postoperative pathology confirmed metastatic GIST with a mitotic count of 6–12 per 5 mm^2^ and clear resection margins (Figure 1F–I). Since the patient declined self-funded mutational analysis, empirical second-line sunitinib therapy (37.5 mg/day) was initiated, followed by routine monitoring. At the last follow-up in January 2025, 39 months post-surgery, no tumor recurrence had been observed. Mild hand-foot syndrome, diarrhea, abdominal pain, and minimal proteinuria had been observed, all of which improved with symptomatic management.

## Discussion

GISTs are usually characterized by gain-of-function mutations in either the *KIT or PDGFRA* genes, leading to constitutive, ligand-independent activation of the respective receptor tyrosine kinases and downstream signaling pathways, resulting in uncontrolled tumor cell proliferation. Imatinib is the standard first-line treatment for unresectable or recurrent metastatic GIST [4]. For GIST with liver metastasis, R0 resection for patients with oligometastases or limited progression during the imatinib response period may improve patient survival [5]. However, liver resection is not recommended for patients with generalized progression or multiple unresectable metastases [6]. Our patient underwent hepatic metastasectomy as limited progression during the imatinib response period was identified. The procedure successfully removed the metastatic tumor, achieving an R0 resection, and alleviated the risk of compression of the porta hepatis. Neoadjuvant therapy was not administered, as the multidisciplinary team determined that expedited surgical intervention was warranted to mitigate imminent hilar compression, given the tumor’s size and anatomical location, prioritizing this over potential tumor volume reduction.

The NCCN Guidelines for GISTs recommend lifelong oral targeted therapy for recurrent or metastatic TKI-sensitive GIST to prevent disease progression. Treatment can be tailored based on imatinib response and mutational testing, with options including continuing imatinib, dose escalation, or switching to second-line therapies. Mutational testing is essential for avoiding ineffective targeted therapies and is a cornerstone of precision GIST management. However, its clinical utility is constrained by global disparities in accessibility, affordability, and healthcare coverage. Even in developed countries like the USA, the mutational testing rate for GIST was only 26.7% among patients diagnosed between 2010 and 2015 [7]. In this case, given our interest in this case’s genotype, we conducted retrospective Sanger sequencing of the resected metastatic tumor during manuscript preparation, revealing coexisting *KIT* exon 11 (p.W557_K558del) and exon 13 (p.V654A) mutations (Figure 1J and K). The *KIT* exon 11 mutation, the most common primary driver, typically confers sensitivity to imatinib, as seen in the patient’s initial response. However, prolonged imatinib use can induce secondary *KIT* mutations, such as the more frequent exon 13 or 14 mutations (in the cytoplasmic ATP-binding domain) or the less frequent exon 17 or 18 mutations (in the activation loop) [8], which are associated with imatinib resistance and disease progression. In this case, empirical imatinib therapy at 600 mg/day was initiated for a 9-cm liver metastasis; in retrospect, 400 mg/day might have been more appropriate, as higher doses are associated with more pronounced side effects and are typically reserved for confirmed resistance or specific mutational profiles, such as KIT exon 9 mutations. Studies show that *KIT* secondary exon 13 or 14 mutations respond better to sunitinib [9], supporting our empirical switch to second-line sunitinib therapy, which was initiated before sequencing data were available. However, other than regorafenib, ripretinib, and avapritinib [10], sunitinib has little activity against secondary exon 17 mutations.

In conclusion, the favorable outcome in this case underscores the clinical utility of sequential TKI therapy combined with elective R0 resection for oligometastatic GIST in the liver. Nonetheless, while retrospective mutational testing supported the postoperative second-line sunitinib regimen, empirical therapy carries an inherent risk of ineffective treatment. Given this limitation, although response-guided empirical approaches may offer practical solutions in real-world resource-limited settings, affordable pretreatment mutational testing—whenever feasible—is strong recommended, which may help to optimize precision oncology strategies for metastatic GIST.

## References

[1] van der Graaf WTA , Tielen R, Bonenkamp JJ et al Nationwide trends in the incidence and outcome of patients with gastrointestinal stromal tumour in the imatinib era. Br J Surg 2018;105:1020–7. 29664995 10.1002/bjs.10809 PMC6033139

[2] DeMatteo RP , Shah A, Fong Y et al Results of hepatic resection for sarcoma metastatic to liver. Ann Surg 2001;234:540–7; discussion 7–8. 11573047 10.1097/00000658-200110000-00013 PMC1422077

[3] Ishizaki M , Uno F, Yoshida R et al Very delayed liver metastasis from small bowel gastrointestinal stromal tumor (32 years after resection of the small bowel GIST): report of a case. Int J Surg Case Rep 2020;76:156–60. 33032047 10.1016/j.ijscr.2020.09.155 PMC7548402

[4] Blanke CD , Demetri GD, von Mehren M et al Long-term results from a randomized phase II trial of standard- versus higher-dose imatinib mesylate for patients with unresectable or metastatic gastrointestinal stromal tumors expressing KIT. J Clin Oncol 2008;26:620–5. 18235121 10.1200/JCO.2007.13.4403

[5] Xue A , Gao X, He Y et al Role of surgery in the management of liver metastases from gastrointestinal stromal tumors. Front Oncol 2022;12:903487. 35847933 10.3389/fonc.2022.903487 PMC9283564

[6] Fairweather M , Balachandran VP, Li GZ et al Cytoreductive surgery for metastatic gastrointestinal stromal tumors treated with tyrosine kinase inhibitors: a 2-institutional analysis. Ann Surg 2018;268:296–302. 28448384 10.1097/SLA.0000000000002281 PMC6203295

[7] Florindez J , Trent J. Low frequency of mutation testing in the united states: an analysis of 3866 GIST patients. Am J Clin Oncol 2020;43:270–8. 31904710 10.1097/COC.0000000000000659

[8] Napolitano A , Vincenzi B. Secondary KIT mutations: the GIST of drug resistance and sensitivity. Br J Cancer 2019;120:577–8. 30792534 10.1038/s41416-019-0388-7 PMC6461933

[9] Heinrich MC , Maki RG, Corless CL et al Primary and secondary kinase genotypes correlate with the biological and clinical activity of sunitinib in imatinib-resistant gastrointestinal stromal tumor. J Clin Oncol 2008;26:5352–9. 18955458 10.1200/JCO.2007.15.7461 PMC2651076

[10] Heinrich MC , Zhang X, Jones RL et al Clinical benefit of avapritinib in KIT-mutant gastrointestinal stromal tumors: a post hoc analysis of the phase I NAVIGATOR and phase I/II CS3007-001 studies. Clin Cancer Res 2024;30:719–28. 38032349 10.1158/1078-0432.CCR-23-1861

